# Supplementary material for: Association between Loss of Immune Checkpoint Programmed Cell Death Protein 1 and Active ANCA-Associated Renal Vasculitis
Source: Int J Mol Sci. 2023 Feb 3;24(3):2975. doi: 10.3390/ijms24032975 (PMC9918235; doi:10.3390/ijms24032975)
Supplement: Supplementary file 1 [file ijms-24-02975-s001.zip › ANCA PD-1_Supplement.pdf]

**Supplemental Table S1. Baseline characteristics of the total cohort.**	
Clinical data	*Value*
Age – years	70 (59-80)
Female sex – no. (%)	10 (66.7)
MPO-/PR3-ANCA – no./no. (%/%)	5/10 (33.3/66.7)
BVAS – points	19 (16-22)
Serum parameters	
Creatinine – mg/dL	3.5 (1.6-4.4)
eGFR – mL/min/1.73 m^2	15.3 (10.5-32.2)
CRP – mg/L	100.2 (33.4-214.3)
Berden classification	
Crescentic class – no. (%)	6 (40)
Focal class – no. (%)	6 (40)
Mixed class – no. (%)	3 (20)
Sclerotic class – no. (%)	0 (0)
ANCA Renal Risk Score	
High risk – no. (%)	1 (6.7)
Intermediate risk – no. (%)	8 (53.3)
Low risk – no. (%)	6 (40)
Tubulointerstitial lesions	
Interstitial inflammation (*i*) – Banff score 0/1/2/3	13/2/0/0
Tubulitis (*t*) – Banff score 0/1/2/3	9/5/1/0
Interstitial fibrosis (*ci*) – Banff score 0/1/2/3	5/3/7/0
Tubular atrophy (*ci*) – Banff score 0/1/2/3	2/9/4/0
Total inflammation (*ti*) – Banff score 0/1/2/3	7/6/2/0
Inflammation in IF/TA (*i-IFTA*) – Banff score 0/1/2/3	5/2/2/6
Tubulitis in IF/TA (*t-IFTA*) – Banff score 0/1/2/3	7/8/0/0
Glomerular lesions	
Normal glomeruli – % of total	42.9 (26.3-92.3)
Glomerular necrosis – % of total	29.4 (7.7-51.1)
Glomerular crescents – % of total	29.4 (7.7-52.6)
Glomerular sclerosis – % of total	5 (0-21.3)
Median (IQR) are shown.	

Supplemental Table S2. Included samples extracted from Nephroseq for glomerular *PDCD1* mRNA expression.			
**Sample name**	**Patient ID**	**Group**	**Glomerular $\log_2$ *PDCD1***
H7Glom.HGU133Plus2.RPGN902.RPGN902	RPGN902	Vasculitis	-1.41453
H7Glom.HGU133Plus2.RPGN904.RPGN904	RPGN904	Vasculitis	-1.18998
H7Glom.HGU133Plus2.RPGN906.RPGN906	RPGN906	Vasculitis	-1.32937
H7Glom.HGU133Plus2.RPGN907.RPGN907	RPGN907	Vasculitis	-0.75724
H7Glom.HGU133Plus2.RPGN908.RPGN908	RPGN908	Vasculitis	-1.01095
H7Glom.HGU133Plus2.RPGN909.RPGN909	RPGN909	Vasculitis	-1.01083
H7Glom.HGU133Plus2.RPGN911.RPGN911	RPGN911	Vasculitis	-0.95146
H7Glom.HGU133Plus2.RPGN913.RPGN913	RPGN913	Vasculitis	-1.2734
H7Glom.HGU133Plus2.RPGN924.RPGN924	RPGN924	Vasculitis	-1.04282
H7Glom.HGU133Plus2.RPGN925.RPGN925	RPGN925	Vasculitis	-0.81824
H7Glom.HGU133Plus2.RPGN926.RPGN926	RPGN926	Vasculitis	-1.15694
H7Glom.HGU133Plus2.RPGN928.RPGN928	RPGN928	Vasculitis	-1.09707
H7Glom.HGU133Plus2.RPGN929.RPGN929	RPGN929	Vasculitis	-1.14146
H7Glom.HGU133Plus2.RPGN930.RPGN930	RPGN930	Vasculitis	-1.02007
H7Glom.HGU133Plus2.RPGN934.RPGN934	RPGN934	Vasculitis	-1.32385
H7Glom.HGU133Plus2.RPGN935.RPGN935	RPGN935	Vasculitis	-0.45529
H7Glom.HGU133Plus2.RPGN936.RPGN936	RPGN936	Vasculitis	-0.66904
H7Glom.HGU133Plus2.RPGN937.RPGN937	RPGN937	Vasculitis	-1.14827
H7Glom.HGU133Plus2.RPGN938.RPGN938	RPGN938	Vasculitis	-0.91126
H7Glom.HGU133Plus2.RPGN939.RPGN939	RPGN939	Vasculitis	-0.82933
H7Glom.HGU133Plus2.RPGN942.RPGN942	RPGN942	Vasculitis	-1.06498
H7Glom.HGU133Plus2.RPGN943.RPGN943	RPGN943	Vasculitis	-1.23878
H7Glom.HGU133Plus2.RPGN946.RPGN946	RPGN946	Vasculitis	-0.71727

Supplemental Table S3. Included samples extracted from Nephroseq for tubulointerstitial *PDCD1* mRNA expression.			
**Sample name**	**Patient ID**	**Group**	**Tubulointerstitial $\log_2$ *PDCD1***
H5Tub.HGU133A.Stanford373.Stanford373	Stanford373	Healthy Living Donor	-0.37886
H5Tub.HGU133A.Stanford374.Stanford374	Stanford374	Healthy Living Donor	-0.19836
H5Tub.HGU133A.Stanford375.Stanford375	Stanford375	Healthy Living Donor	-0.32156
H5Tub.HGU133A.Stanford378.Stanford378	Stanford378	Healthy Living Donor	0.19122
H7Tub.HGU133Plus2.Stanford1164.Stanford1164	Stanford1164	Healthy Living Donor	0.31434
H7Tub.HGU133Plus2.Stanford1165.Stanford1165	Stanford1165	Healthy Living Donor	-0.56408
H7Tub.HGU133Plus2.Stanford1166.Stanford1166	Stanford1166	Healthy Living Donor	-0.33527
H7Tub.HGU133Plus2.Stanford1168.Stanford1168	Stanford1168	Healthy Living Donor	-0.22082
H7Tub.HGU133Plus2.Stanford1169.Stanford1169	Stanford1169	Healthy Living Donor	-0.36906
H7Tub.HGU133Plus2.Stanford1170.Stanford1170	Stanford1170	Healthy Living Donor	-0.24186
H7Tub.HGU133Plus2.Stanford1171.Stanford1171	Stanford1171	Healthy Living Donor	-0.47705
H7Tub.HGU133Plus2.Stanford1172.Stanford1172	Stanford1172	Healthy Living Donor	-0.40891
H7Tub.HGU133Plus2.Stanford1173.Stanford1173	Stanford1173	Healthy Living Donor	-0.06379
H7Tub.HGU133Plus2.Stanford1174.Stanford1174	Stanford1174	Healthy Living Donor	-0.09738
H7Tub.HGU133Plus2.Stanford1175.Stanford1175	Stanford1175	Healthy Living Donor	0.12195
H7Tub.HGU133Plus2.Stanford1177.Stanford1177	Stanford1177	Healthy Living Donor	-0.09665
H7Tub.HGU133Plus2.Stanford1178.Stanford1178	Stanford1178	Healthy Living Donor	-0.24309
H7Tub.HGU133Plus2.Stanford1179.Stanford1179	Stanford1179	Healthy Living Donor	-0.09855
H7Tub.HGU133Plus2.Stanford1180.Stanford1180	Stanford1180	Healthy Living Donor	-0.30467
H7Tub.HGU133Plus2.Stanford1182.Stanford1182	Stanford1182	Healthy Living Donor	-0.12465
H7Tub.HGU133Plus2.Stanford1183.Stanford1183	Stanford1183	Healthy Living Donor	-0.3736
H7Tub.HGU133Plus2.Stanford1184.Stanford1184	Stanford1184	Healthy Living Donor	-0.37375
H8Tub-HGU133Plus2-LD_Stanford1296-LD_Stanford1296_30_25	Stanford1296	Healthy Living Donor	-0.75229
H8Tub-HGU133Plus2-LD_Stanford1298-LD_Stanford1298_30_103	Stanford1298	Healthy Living Donor	-0.34307
H8Tub-HGU133Plus2-LD_Stanford1299-LD_Stanford1299_30_109	Stanford1299	Healthy Living Donor	-0.30882
H8Tub-HGU133Plus2-LD_Stanford1301-LD_Stanford1301_30_131	Stanford1301	Healthy Living Donor	-0.31042
H8Tub-HGU133Plus2-LD_Stanford1302-LD_Stanford1302_30_138	Stanford1302	Healthy Living Donor	-0.27991
H8Tub-HGU133Plus2-LD_Stanford1303-LD_Stanford1303_30_142	Stanford1303	Healthy Living Donor	-0.29704
H8Tub-HGU133Plus2-LD_Stanford1304-LD_Stanford1304_30_146	Stanford1304	Healthy Living Donor	-0.62814
H8Tub-HGU133Plus2-LD_Stanford1305-LD_Stanford1305_30_147	Stanford1305	Healthy Living Donor	-0.22632
H8Tub-HGU133Plus2-LD_Stanford1306-LD_Stanford1306_30_150	Stanford1306	Healthy Living Donor	-0.3535
H7Tub.HGU133Plus2.RPGN1102.RPGN1102	RPGN1102	Vasculitis	-0.36285
H7Tub.HGU133Plus2.RPGN1103.RPGN1103	RPGN1103	Vasculitis	-0.48674
H7Tub.HGU133Plus2.RPGN1106.RPGN1106	RPGN1106	Vasculitis	-0.64861
H7Tub.HGU133Plus2.RPGN1107.RPGN1107	RPGN1107	Vasculitis	-0.29265
H7Tub.HGU133Plus2.RPGN1109.RPGN1109	RPGN1109	Vasculitis	-0.63569
H7Tub.HGU133Plus2.RPGN1113.RPGN1113	RPGN1113	Vasculitis	-0.31303
H7Tub.HGU133Plus2.RPGN1118.RPGN1118	RPGN1118	Vasculitis	-0.5105
H7Tub.HGU133Plus2.RPGN1119.RPGN1119	RPGN1119	Vasculitis	-0.87159
H7Tub.HGU133Plus2.RPGN1120.RPGN1120	RPGN1120	Vasculitis	-0.77136
H7Tub.HGU133Plus2.RPGN1121.RPGN1121	RPGN1121	Vasculitis	-0.44042
H7Tub.HGU133Plus2.RPGN1122.RPGN1122	RPGN1122	Vasculitis	-0.46473
H7Tub.HGU133Plus2.RPGN1123.RPGN1123	RPGN1123	Vasculitis	-0.31426
H7Tub.HGU133Plus2.RPGN1126.RPGN1126	RPGN1126	Vasculitis	-0.80338
H7Tub.HGU133Plus2.RPGN1127.RPGN1127	RPGN1127	Vasculitis	-0.47247
H7Tub.HGU133Plus2.RPGN1128.RPGN1128	RPGN1128	Vasculitis	-0.5087
H7Tub.HGU133Plus2.RPGN1129.RPGN1129	RPGN1129	Vasculitis	-0.32424
H7Tub.HGU133Plus2.RPGN1130.RPGN1130	RPGN1130	Vasculitis	-0.20402
H7Tub.HGU133Plus2.RPGN1131.RPGN1131	RPGN1131	Vasculitis	-0.40236
H7Tub.HGU133Plus2.RPGN1134.RPGN1134	RPGN1134	Vasculitis	-0.45873
H7Tub.HGU133Plus2.RPGN1135.RPGN1135	RPGN1135	Vasculitis	-0.50688
H7Tub.HGU133Plus2.RPGN1138.RPGN1138	RPGN1138	Vasculitis	-0.20519
